# Short- and Long-Term Outcomes of Coronary Artery Bypass Grafting with or without the Bypass Grafting Located in the Chronically Occluded Right Coronary Artery

**DOI:** 10.21470/1678-9741-2023-0390

**Published:** 2026-05-06

**Authors:** Changcheng Liu, Ying Du, Hui Li, Haiyang Li, Chengxiong Gu

**Affiliations:** 1 Department of Cardiac Surgery, Beijing Anzhen Hospital, Capital Medical University, Beijing, People’s Republic of China

**Keywords:** Right Coronary Artery, Chronic Total Occlusion, Coronary Artery Bypass Grafting, Clinical Outcomes.

## Abstract

**Introduction:**

Chronic total occlusion (CTO) in the right coronary artery (RCA) is common in multivessel coronary artery disease. The impact of bypass grafting in the small target vessel (1.0-1.5 mm) of the RCA-CTO on outcomes is unknown.

**Methods:**

RCA-CTO was treated with either bypass grafting (RCA-bypass group) or non-bypass grafting (non-RCA-bypass group). The study compared the shortand long-term outcomes of patients who underwent coronary artery bypass grafting with or without the bypass grafting located in the small target vessel of the RCA-CTO.

**Results:**

A total of 426 patients were enrolled in the present study, including 376 patients in the RCA-bypass group and 50 patients in non-RCA-bypass group. The 30-day all-cause death in the RCA-bypass and non-RCA-bypass groups was 2.39% and 2.0% (P=0.999), respectively. The median follow-up time was 73 months. The long-term major adverse cardiac and cerebrovascular events in the RCA-bypass and non-RCA-bypass groups were 6.91% and 18% (P=0.013), respectively. There was a trend toward a higher rate of long-term freedom from major adverse cardiac and cardiovascular events within the RCA-bypass group (log-rank P=0.088).

**Conclusions:**

The bypass grafting located in the small target vessel of the RCA-CTO did not affect the short-term outcomes, but it was associated with a reduced risk of long-term major adverse cardiac and cardiovascular events in patients who underwent coronary artery bypass grafting.

## INTRODUCTION

**Table:** 

Abbreviations, Acronyms & Symbols				
ACEI	= Angiotensin-converting enzyme inhibitor		LIMA	= Left internal mammary artery
AMI	= Acute myocardial infarction		LVEF	= Left ventricular ejection fraction
ARB	= Angiotensin receptor inhibitor		MACCEs	= Major adverse cardiac and cerebrovascular events
BMI	= Body mass index		MI	= Myocardial infarction
CABG	= Coronary artery bypass grafting		OM	= Oblique marginal artery
CAD	= Coronary artery disease		PCI	= Percutaneous coronary intervention
CCB	= Calcium channel blocker		PDA	= Posterior descending artery
CCS	= Canadian Cardiovascular Society		PLV	= Posterior branch of left ventricle
CE	= Coronary endarterectomy		PVD	= Peripheral vascular disease
CI	= Confidence interval		RCA	= Right coronary artery
CTO	= Chronic total occlusion		RUMAS	= Ramus intermedius artery
HR	= Hazards ratio		SV	= Saphenous vein
IABP	= Intra-aortic balloon pump		SVG	= Saphenous vein graft
ICU	= Intensive care unit		T2DM	= Type 2 diabetes mellitus
LAD	= Left anterior descending artery			

%-50% of patients with CTO^[1^-^3]^. Coronary artery bypass grafting (CABG) successfully achieves revascularization in approximately 90% of patients with CTO^[4]^. However, surgical revascularization failure is more frequently observed in the RCA-CTO than in the left anterior descending artery (LAD)^[5]^.

Target coronary arteries for bypass in RCA-CTO are often concomitant with diffuse lesions, narrow lumen (< 1.5 mm), or severe calcification, which increase the treatment’s technical difficulty. Moreover, collateral circulation is frequently observed in RCA-CTO, which is usually associated with reducing acute myocardial infarction (AMI) and improving survival^[6]^. However, there is limited evidence regarding survival benefit from bypass grafting in the small target vessel (1.0-1.5 mm) of the RCA-CTO in patients who underwent CABG. Thus, the present study compared the shortand long-term outcomes of patients who underwent CABG with or without the bypass grafting located in the small target vessel of the RCA-CTO.

## METHODS

### Study Definitions

The CTO lesion was defined as a complete occlusion of coronary artery with grade 0 thrombolysis in myocardial infarction flow for at least three months. The small target vessel was defined as 1.0-1.5 mm of the reference vessel diameter.

The coronary collateral circulation was graded based on the Rentrop collateral classification scale^[7]^ as follows: grade 0, no collateral filling; grade 1, filling of side branches without epicardial segment of recipient vessel; grade 2, partial filling of the recipient epicardial segment through collateral vessels; and grade 3, complete collateral filling of the epicardial vessel segment to be dilated. Grades 2 and 3 were defined as well-developed collateral circulation.

RCA-CTO with small target vessels was defined as a complete occlusion of right main coronary artery and the major branches of posterior descending artery and posterior branch of left ventricle with 1.0-1.5 mm of lumen diameter.

### Study Population and Grouping

Between January 2012 and December 2021, 426 patients who were received off-pump CABG were included in this retrospective study. The inclusion criteria were as follows: 1) age > 18 years; 2) right dominant type of coronary artery classification; 3) three-vessel coronary artery disease (CAD) with RCA-CTO with small target vessels; and 4) scheduled CABG. The exclusion criteria were as follows: 1) myocardial infarction (MI) within 30 days due to culprit vessel in RCA; 2) RCA with non-CTO disease and diameter of target vessel > 1.5 mm; (3) urgent or emergent CABG; and (4) concomitant valvular or aortic surgery.

According to whether or not the bypass graft was located in the RCA territory, the patients were divided into the RCA-bypass group (n=376) and the non-RCA-bypass group (n=50). All patients in both groups received optimal medical therapy after surgery. In brief, dual antiplatelet therapy (aspirin + clopidogrel or ticagrelor) within one year and single antiplatelet therapy (aspirin or clopidogrel) after one year as well as a lipid lowering therapy and other secondary preventions were recommended to the patients as needed according to the CAD guidelines^[8]^.

The present study was approved by the Ethics Committee of our institution (2020050X) and was performed following the Ethical Guidelines of the Committee on Human Experimentation of our institution. Informed consent was obtained from all patients.

### Surgical Procedure

Off-pump CABG was performed with a standard procedural protocol involving general anesthesia, median sternotomy, systemic heparinization with activated clotting time > 300 s, and routine harvesting of the left internal mammary artery (LIMA) and saphenous vein (SV). Bypass grafting was performed with the assist of an octopus cardiac stabilizer. LIMA was always anastomosed to the LAD. The SV graft was performed by proximal anastomosis to the ascending aorta first and then distal anastomosis to the left circumflex coronary artery and RCA in a sequential manner. The intraoperative quality of the bypass graft was measured quantitatively by transit-time flow measurement using the VeriQ system device (MediStim Inc., Oslo, Norway). Graft patency was defined by a mean graft flow > 20 ml/min and pulsatility index < 5 when the blood pressure was > 90/60 mmHg.

The anastomosis techniques of RCA included end-to-side anastomosis, side-to-side anastomosis, and coronary endarterectomy (CE) with end-to-side anastomosis.

CE was performed when no suitable anastomotic location was present in the middle-distal segment of the coronary artery with a wide blood supply territory. The atherosclerotic plaque was removed using the closed CE technique with the following steps: 1) make a 5-8 mm arteriotomy at the anastomotic site; 2) dissect the anatomy gap between the adventitia and mature plaque using Potts scissors from both sides of the incision; and 3) remove plaque with the help of cardiac contraction reaction force. The distal end of the plaque was completely dissected, and the proximal end of the plaque was dissected 3-5 mm and then cut off to avoid competitive flow. A satisfactory closed CE included a translucent and rat-tail shape at the distal end of the plaque and reverse blood flow from the anastomotic site.

The side-to-side anastomosis technique was performed when the diameter mismatch between the saphenous vein graft (SVG) and RCA (SVG-RCA diameter ratio) was ≥ 4:1. The details of this technique have been previous reported^[9]^.

### Study Endpoints

The primary endpoints were 30-day all-cause death and long-term major adverse cardiac and cerebrovascular events (MACCEs), including MI, stroke, death, and redo revascularization.

Secondary endpoints included duration of mechanical ventilation, duration of intensive care unit (ICU) stay, mechanical circulation support, recurrence of angina (Canadian Cardiovascular Society [CCS] grade III or IV), and long-term survival. Follow-up was mainly performed by outpatient clinic visit and telephone interviews, and it ended in December 2021.

### Statistical Analysis

Data analysis was performed using SAS software (version 9.4; SAS Institute Inc., Cary, North Carolina, United States of America). Data are presented as the mean ± standard deviation or the median with interquartile range for continuous variables. Data are presented as frequencies and percentages for categorical variables. Analysis of variance was used to assess non-paired samples for the comparison of normally distributed parameters, and the Wilcoxon rank-sum test was used for the comparison of non-parametric variables. The Chi-squared test and Fisher’s exact test were applied for the comparison of categorical variables. Kaplan-Meier survival analysis was performed for long-term survival. The Cox proportional hazards model was used to identify the effect of RCA-CTO revascularization on long-term MACCEs. Differences were considered statistically significant when the *P*-value was < 0.05.

## RESULTS

### Demographics and Comorbidities

The demographics, cardiac parameters, and comorbidities of the two groups were similar. The number of grafts in the RCA bypass group was significantly higher than that in the non-RCA bypass group (3.80 ± 0.57 *vs.* 3.42 ± 0.57, *P*<0.001). Medical therapy after surgery was not different between the two groups. The demographics, cardiac parameters, comorbidities, surgical parameters, and medicine at discharge in both groups are summarized in Table 1.

**Table 1:** Baseline and operative characteristics.

Variables	RCA-bypass group	Non-RCA-bypass group	*P*-value
	(n=376)	(n=50)	
Age (years)	61.04 ± 8.99	61.82 ± 9.40	0.274
Male sex	300 (79.79)	37 (74.00)	0.344
BMI (kg/m^2^)	25.93 ± 2.87	26.69 ± 2.83	0.099
Hypertension	228 (60.64)	26 (52.00)	0.242
Diabetes mellitus	145 (38.56)	21 (42.00)	0.640
PVD	115 (30.59)	15 (30.00)	0.934
Prior stroke	48 (12.77)	8 (16.00)	0.507
Prior MI	27 (7.18)	4 (8.00)	0.774
Prior PCI	26 (6.91)	2 (4.00)	0.759
CCS angina class			0.58
I or II	241 (64.10)	34 (68.00)	
III or IV	135 (35.90)	16 (32.00)	
LVEF (%)	61.03 ± 7.47	61.71 ± 8.32
LVEF < 50%	45 (11.97)	8 (16)	0.463
Rentrop collateral classification scale of RCA-CTO			0.142
Grade 0-1	96 (25.53)	10 (20.00)	
Grade 2-3	280 (74.47)	40 (80.00)	
Number of grafts	3.80 ± 0.57	3.42 ± 0.57	< 0.001
LIMA-LAD grafting	291 (77.39)	37 (74.00)	0.592
Target vessel for grafting			
LAD	376 (100.00)	50 (100.00)	0.999
Diagonal artery	282 (75.00)	33 (66.00)	0.174
RUMAS	41 (10.90)	3 (6.00)	0.456
OM	282 (75.00)	41 (82.00)	0.277
PLV	83 (22.07)	10 (20.00)	0.739
PDA	287 (76.33)	-	-
RCA	82 (21.81)	-	-
RCA anastomosis			
End-to-side	199 (52.92)	-	-
CE + end-to-side	159 (42.29)	-	-
Side-to-side	18 (4.79)	-	-
Medicine at discharge			
Aspirin	368 (97.87)	49 (98.00)	0.999
Ticagrelor	127 (33.78)	14 (28.00)	0.415
Clopidogrel	236 (62.76)	34 (68.00)	0.274
β-blocker	274 (72.87)	39 (78.00)	0.440
Statins	372 (98.94)	49 (98.00)	0.999
CCB	158 (42.02)	23 (46.00)	0.593
ACEI or ARB	207 (55.05)	29 (58.00)	0.694

ACEI=angiotensin-converting enzyme inhibitor; ARB=angiotensin receptor inhibitor; BMI=body mass index; CCB=calcium channel blocker; CCS=Canadian Cardiovascular Society; CE=coronary endarterectomy; CTO=chronic total occlusion; LAD=left anterior descending artery; LIMA=left internal mammary artery; LVEF=left ventricular ejection fraction; MI=myocardial infarction; OM=oblique marginal artery; PCI=percutaneous coronary intervention; PDA=posterior descending artery; PLV=posterior branch of left ventricle; PVD=peripheral vascular disease; RCA=right coronary artery; RUMAS=ramus intermedius artery

### Short-Term Outcomes

The 30-day all-cause mortality was similar in both groups (RCA-bypass group, 2.39%; and non-RCA-bypass group, 2.0%; *P*=0.999). The duration of mechanical ventilation, duration of ICU stay, and mechanical circulation support did not significantly differ between the two groups. The details of the 30-day outcomes are shown in Table 2.

**Table 2:** The 30-day outcomes and long-term MACCEs.

Variables	RCA-bypass group	Non-RCA-bypass group	*P*-value
	(n=376)	(n=50)	
30-day outcomes			
All-cause death	9 (2.39)	1 (2.00)	0.999
IABP assistance	25 (6.65)	3 (6.00)	0.999
Duration of ventilation (hours)	16 (9.20)	14 (8,19)	0.354
ICU stay (days)	1 (1,2)	1 (1,2.5)	0.068
Long-term outcomes			
MI	2 (0.53)	0 (0)	0.999
All-cause death	15 (3.99)	6 (12.00)	0.026
Cardiac death	6 (1.60)	2 (4.00)	0.220
Non-cardiac death	9 (2.39)	4 (8.00)	0.054
Stroke	5 (2.42)	1 (5.26)	0.413
Revascularization	5 (1.33)	2 (4.00)	0.193
MACCEs	26 (6.91)	9 (18.00)	0.013
Angina recurrence (CCS class III or IV)	25 (6.65)	7 (14.00)	0.082

CCS=Canadian Cardiovascular Society; IABP=intra-aortic balloon pump; ICU=intensive care unit; MACCEs=major adverse cardiac and cerebrovascular events; MI=myocardial infarction; RCA=right coronary artery

### Long-Term Outcomes

The median duration of follow-up was 73 (22, 90) months. Moreover, the incidence of MI and cardiac death was similar in both groups. Angina recurrence (CCS class III or IV) in the non-RCA-bypass group was numerically higher than in the RCA-bypass group, but there was no statistical difference (6.5% *vs.* 14%, *P*=0.082).

All-cause death in the non-RCA-bypass group was significantly higher than in the RCA-bypass group (3.99% *vs.* 12%, respectively; *P*=0.026). The cumulative incidence of MACCEs in the RCA-bypass and non-RCA-bypass groups was 6.91% and 18%, respectively (*P*=0.013). The long-term outcomes are summarized in Table 2.

**Fig. 1:**
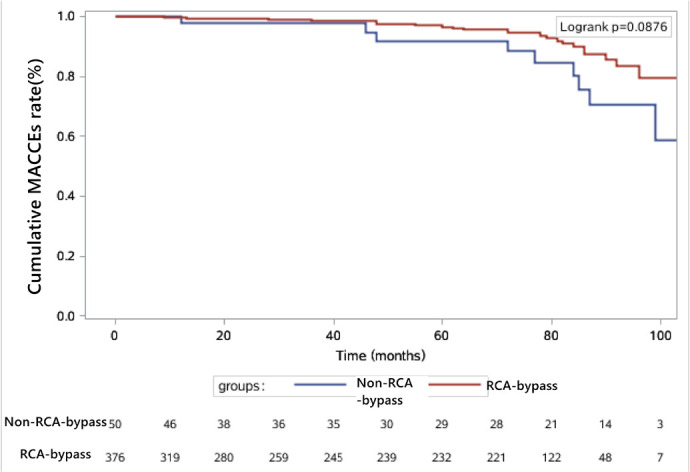
Kaplan-Meier survival analysis of long-term freedom from major adverse cardiac and cerebrovascular events (MACCEs) in two groups. RCA=right coronary artery.

The multivariate Cox regression analysis of the subgroups (Table 3) indicated that bypass grafting in the RCA significantly reduced the risk of long-term MACCEs (hazards ratio [HR]=0.410, 95% confidence interval [CI]: 0.177-0.948, *P*=0.037)] and that peripheral vascular disease was an independent risk factor (HR=2.430, 95% CI: 1.219-4.842, *P*=0.012). Kaplan-Meier survival analysis (Figure 1) indicated that there was a trend toward a higher rate of long-term freedom from MACCEs within the RCA bypass group, but this trend did not reach statistical significance (log-rank *P*=0.088).

**Table 3:** Cox regression analysis to identify risk factors of long-term MACCEs.

Variables	HR	95% CI	*P*-value
Bypass in RCA	0.410	0.177-0.948	0.037
Age > 65 years	1.297	0.633-2.661	0.477
BMI	0.898	0.795-1.014	0.082
Female	0.360	0.108-1.203	0.097
Hypertension	1.017	0.485-2.135	0.964
T2DM	0.801	0.379-1.694	0.562
PVD	2.430	1.219-4.842	0.012

BMI=body mass index; CI=confidence interval; HR=hazards ratio; MACCEs=major adverse cardiac and cerebrovascular events; PVD=peripheral vascular disease; RCA=right coronary artery; T2DM=type 2 diabetes mellitus

## DISCUSSION

### Major Findings

The bypass grafting located in the small target vessel of the RCA-CTO did not affect short-term outcomes of CABG. But patients who underwent bypass grafting in the RCA-CTO had lower risk of long-term all-cause death and MACCEs.

### Residual Myocardial Ischemia Despite Well-Developed Collateral Circulation

Well-developed collateral circulation may be a protective factor in patients with CTO and may improve freedom from MI. Claessen et al.^[10]^ reported that successful percutaneous coronary intervention (PCI) for RCA-CTO does not offer survival benefits, which supports this hypothesis. However, Konstanty-Kalandyk et al.^[11]^ reported that RCA-CTO increases the risk of MACCEs in patients undergoing CABG. Moreover, nuclear imaging by positron emission tomography has indicated that there is no significant correlation between the severity of perfusion deficit and the collateral grade on myocardial viability assessment in patients with coronary CTO and collateral circulation^[12]^. Therefore, these studies indicate a weak relationship between well-developed collateral circulation and myocardial protection.

Several studies have reported that regardless of PCI or CABG, aggressive revascularization reduces the risk of long-term major adverse cardiovascular events compared to medical therapy alone in patients with coronary CTO and well-developed collateral circulation^[13^-^15]^. In line with previous studies, our results also suggested that restoring antegrade coronary flow by CABG, even in patients with small RCA-CTO and collateral circulation, may improve myocardial blood supply and, thus, long-term survival. And the multivariate Cox regression analysis also indicated that bypass grafting in the RCA-CTO was a protective factor to reduce approximately three times all-cause death compared with non-grafting in the RCA-CTO. Furthermore, for death analysis, the cardiac death and non-cardiac death in the RCA bypass group were lower than in the non-RCA-bypass group, but it did not achieve a statistical difference, mainly due to the small sample size of the non-RCA-bypass group.

### Bypass Grafting Tactics in the Small Target Vessel of RCA-CTO

In general, cardiac surgeons perform a bypass grafting in a CTO vessel based on angiographic findings (degree of collateralization and Rentrop grade) and intraoperative visual exploration (caliber, degree of calcification, and run-off).

The SYNTAX studies^[16^,^17]^ have reported that patients with CTO are less likely to achieve complete revascularization in the PCI and CABG groups due to CTO vessels being smaller in size and having diffuse lesions.

CE is a proven surgical technique for complete myocardial revascularization in CAD with diffuse lesions^[18^,^19]^. In the present study, 42.29% of patients in the RCA-bypass group received CE. Completely removing plaques is critical to maintain good run-off, and reendothelialization after CE reduces the risk of restenosis and thrombosis as well as improves long-term prognosis^[20^,^21]^.

CE mainly increases the risk of acute thrombosis-related AMI due to the activation of platelets and the coagulation cascade caused by removing plaques^[22]^. Recent advancements in antithrombotic agents and perioperative care management have reduced the risk of morbidity and mortality in patients who have undergone CE^[23]^.

The SVG is widely used in CABG, especially in RCA. However, the patency of SVG grafted to RCA is poor, and diameter mismatch between SVG and RCA is an important cause of graft failure. Yamane et al.^[24]^ reported that an SVG-RCA diameter ratio ≤ 2.8 improves the three-year patency rate. Therefore, selection of matched SVG to minimize caliber mismatch is reasonable, especially in small target vessels. Furthermore, we have previously reported that bypass grafting in small target vessels using side-to-side anastomosis produces favorable hemodynamic flow patterns compared to end-to-side anastomosis^[9]^. Computational fluidic dynamics models have also indicated that side-to-side anastomosis results in a smoother blood flow and smaller spatial gradients of wall shear stress compared to end-to-side anastomosis^[25]^.

Moreover, SVG, as a sequential graft, bypassed to RCA territory shows superior long-term patency compared to individual grafting to RCA territory^[26]^.

### Limitations

The present study had several limitations. First, the retrospective and observational design was susceptible to inherent bias. However, this is the first report specifically addressing the issue of bypass grafting in small target vessels as well as evaluating the potential long-term survival benefit in RCA-CTO patients.

Second, the number of patients in the non-RCA-bypass group was small. Sample size mismatch may have impacted the statistical power. The univariate analysis indicated that bypass grafting in RCA-CTO reduced MACCEs, but the survival analysis indicated that long-term MACCEs was not significantly different between the two groups. These differences may be related to follow-up duration and unmeasured confounders. Thus, multivariate Cox proportional hazards was used to adjust for confounding factors and to further assess the robustness of our findings. And further study with large sample size should be conducted to support our findings.

Third, coronary angiography was not part of the routine follow-up. Therefore, coronary artery computed tomographic angiography will be needed to assess graft patency in small target vessels in the future.

## CONCLUSION

The present findings demonstrated that for RCA-CTO with a small target vessel, patients who underwent CABG with or without the bypass grafting located in RCA have similar short-term outcomes. But bypass grafting in the small target vessel of the RCA-CTO is associated with a reduced risk of long-term MACCEs.

## Data Availability

The data supporting the findings of this study are available from the corresponding author upon reasonable request via e-mail.

## Artificial Intelligence Usage

The authors declare that no artificial intelligence tools were used in the preparation, writing, or editing of this manuscript. The authors take full responsibility for the content to be published.
